# The properties of the positronium lifetime image reconstruction based on maximum likelihood estimation

**DOI:** 10.5604/01.3001.0054.1807

**Published:** 2023-12-30

**Authors:** Zhuo Chen, Lingling An, Chien-Min Kao, Hsin-Hsiung Huang

**Affiliations:** 1 Department of Mathematics, University of Arizona, Tucson, USA; 2 Department of Agricultural and Biosystems Engineering, University of Arizona, Tucson, USA; 3 Department of Radiology, University of Chicago, Chicago, USA; 4 Department of Statistics and Data Science, University of Central Florida, Orlando, USA

**Keywords:** error bounds, imaging reconstruction, positronium lifetime decay

## Abstract

The positronium lifetime imaging (PLI) reconstruction is a technique used in time-of-flight (TOF) positron emission tomography (PET) imaging that involves measuring the lifespan of positronium, which is a metastable electron-positron pair that arises when a PET molecule releases a positron, prior to its annihilation. We have previously developed a maximum likelihood (ML) algorithm for PLI reconstruction and demonstrated that it can generate quantitatively accurate lifetime images for a 570 ps (pico-seconds) TOF PET system. In this study, we conducted further investigations into the statistical properties of the algorithm, including the variability of the reconstruction results, the sensitivity of the algorithm to the number of acquired PLI events and its robustness to hyperparameter choices. Our findings indicate that the proposed ML method produces sufficiently stable lifetime images to enable reliable distinction of regions of interest. Moreover, the number of PLI events required to produce quantitatively accurate lifetime images is computationally plausible. These results demonstrate the potential of our ML algorithm for advancing the capabilities of TOF PET imaging.

## INTRODUCTION

Positron emission tomography (PET [1]) has been extensively utilised in the diagnosis of diseases such as cancer and Alzheimer’s disease. The method relies on the uptake of radiolabelled molecules that specifically target pathological signatures associated with these diseases. Recently, Moskal et al. introduced a new concept of imaging with PET called positronium imaging [2–4] and demonstrated ex-vivo positronium lifetime images by using the J-PET system [5]. Positronium is a metastable electron-positron pair that may be formed prior to annihilation of a positron after it is released by a PET molecule [6, 7]. Positronium imaging refers to the determination of images of the positronium properties [2]. It includes the imaging of the ratio of positronium decay rates into three and two photons (three-gamma/two-gamma rate ratio) and positronium lifetime imaging (PLI) that measures the lifespan of the positronium. In living organisms, these positronium properties can reflect certain conditions of the tissue microenvironment, adding information to that given by PET tracer uptake.

According to the history of the positron, the positron lifetime spectrum can be decomposed into several components, including those corresponding to formations of the so-called ortho-positronium and para-positronium. It was shown in recent studies that the ortho-positronium lifetime in healthy adipose tissue differs from the ortho-positronium lifetime in cardiac myxoma tumours [8]. Ex-vivo PLI has until now been based on using the back-projection (BP) method for image reconstruction. As the current time-of-flight (TOF) PET systems have a resolution in the range of 200–600 ps FWHM [9–11], the resulting BP-based PLI images have poor resolution and are quantitatively inaccurate. The development of PLI reconstruction methods for TOF PET systems having finite TOF resolution is therefore of interest.

In this article, we focus on the lifetime of ortho-positronium because it is the component that is sensitive to the tissue microenvironment. To date, aside from our method [12], the idea of reconstructing uptake and lifetime images from PLI data has only been investigated by Qi and Huang [13] and Shopa and Dulski [14]. In their studies, the lifetime measurement does not include the effects of finite time resolution of the detectors or the difference in the flight time of the gamma rays prior to detection. In this article, we will include the former. Furthermore, the focus of the previous studies is to demonstrate PLI for a reasonable number of events. As PLI measurement involves triple-coincidence detection that has a low sensitivity, a focus of this article is to examine the minimum number of events that is sufficient for producing reasonable PLI images.

In this work, we investigate the properties the maximum likelihood estimator of the positronium lifetime imaging reconstruction, with the goals of examining the accuracy of the proposed algorithm by considering Gaussian blur, evaluating the stability of the PLI reconstruction by performing multiple replications for each simulation setting, and assessing the impact of the uncertainty in time measurements and the sample size of PLI events on the performance of the proposed method.

## METHODS

The methods applied in this study follow our previous work [12]. We assume that τ, the lifespan of a positron prior to its annihilation, follows an exponential distribution with a density function

(1) $$f(\tau ;\lambda )=\lambda e^{-\lambda \tau },$$

The conventional TOF PET system reports the index of the two detectors hit by the two opposite 511 keV gamma rays, denoted as $i_{511keV,1}\;i_{511keV,2}$ and the TOF defined as

(2) $$\begin{array}{l} {\Delta t}_{511keV}=t_{511keV,1}-t_{511keV,2}\\ \;=\frac{\alpha_{511keV,1}-\alpha_{511keV,2}}{v^{c}}, \end{array}$$

where $i_{511}keV,1$ and $i_{511}keV,2$ denote the detection times of the two opposite 511 keV gamma rays, and $\alpha_{511}keV,1$ and $\alpha_{511}keV,2$ are the travelling distances of these photons from the decay position to the detectors.

We assume that the system is extended further to allow triple coincidence detection and also to report iγ, the index of the detector hit by the prompt gamma ray, and Δtγ, defined as

(3) $$\Delta t_{\gamma }=\frac{t_{511\mathrm{keV},1}+t_{511\mathrm{keV},2}}{2}-t_{\gamma }.$$

Suppose that an isotope decay occurs at time $t_{\text{decay}}$, we have

(4) $$t_{511\mathrm{keV},1}=t_{\text{decαy}}+\frac{\alpha_{511\mathrm{keV},1}}{\upsilon_{c}},$$

(5) $$t_{511\mathrm{keV},2}=t_{\text{decαy}}+\frac{\alpha_{511\mathrm{keV},2}}{\upsilon_{c}},$$

and

(6) $$t_{\gamma }=t_{dec\alpha y}+\frac{\alpha_{\gamma }}{\upsilon_{c}},$$

where $\alpha_{\gamma }$ is the travelling distance of the prompt gamma ray.

Plugging Eqs. (4)–(6) into Eq. (3) and rearranging the resulting equation, we obtain

(7) $$\tau =\Delta t_{\gamma }-\frac{\alpha_{511\mathrm{keV},1}+\alpha_{511\mathrm{keV},2}-2\alpha_{\gamma }}{2\upsilon_{c}}.$$

In this paper, we will use the known value of the second term on the righthand side to obtain the measured lifespan $\tau^{\wedge }$ from the simulated $\Delta t_{\gamma }$.

We assume that the uncertainty in time measurement by all detectors is identical and independent, modelled by a one-Xdimensional (1-d) Gaussian distribution $N\left(0,\sigma_{t}\right)$. Then, from Eqs. (2) and (3), we have $\sigma \Delta t_{511\mathrm{keV}}=\surd 2\sigma_{t}$ and $\Delta t\gamma =\surd 3/2\sigma_{t}$. Under this assumption, the resolution in therefore equals the TOF resolution of the system multiplied by √3/2. Since τ follows an exponential distribution and Δtγ follows a Gaussian distribution given τ,Δtγ and hence follow an Exponentially Modified Gaussian distribution [15] EMG(μ,λ,σ), where μ and σ are the mean and standard deviation of the Gaussian distribution, and λ is the parameter of the exponential distribution. As discussed, for $\overset{ˆ}{\tau }$ we have

$$\mu =0\;\text{and}\;\sigma =\surd 3/2\sigma_{t}.$$

The maximum likelihood estimate (MLE) is based on the log-likelihood of (λ,f) introduced in [12], with the observed positronium lifespan $\overset{ˆ}{\tau }$ following an EMG distribution, γ-ray activities (photon counts that follow a Poisson distribution of mean f), and the system matrix $H=\left[H_{c,j}\right]$ which gives the probability that a positron decay occurring inside image pixel j would give rise to an event at line of response (LOR) c. Here, $\lambda =\left[\lambda_{j}\right]$ and $f=\left[f_{j}\right]$ denote the lifetime and activity image respectively. We derive the maximum likelihood estimation (MLE) based on the true τ, as done in [13], and denote the PLI list mode (LM) data as $W_{Nk}^{0}={\left\{w_{k}^{0}\right\}}_{k=1}^{N_{k}}$

where $N_{k}$ is the total number of events acquired. The MLE of λ based on the profile log-likelihood of (λ,) given $W_{N_{k}}^{0}$ and the MLE of f based on the marginal log-likelihood of f are given as

$$\begin{array}{l} arg\;\underset{\lambda }{max}\;\ell (\lambda ;\overset{ˆ}{f},{𝒲}_{N_{k}}^{0})\\ \;=arg\;\underset{\lambda }{max}\;\sum_{k=1}^{N_{k}}\;ln\left(\sum_{j=1}^{N_{j}}\;H_{c_{k}j}{\overset{ˆ}{f}}_{j}EMG\left(\tau_{k};\lambda_{j},\sigma^{2}\right)\right) \end{array}$$

and

$$\begin{array}{l} arg\;\underset{f}{max}\;\ell \left(f;{𝒞}_{N_{k}}\right)\\ \;=arg\;\underset{f}{max}\;\sum_{k=1}^{N_{k}}\;ln\left(\sum_{j=1}^{N_{j}}\;H_{c_{k},j}f_{j}\right) \end{array}$$

where $N_{j}$ is the number of image pixels, and the exponentially modified Gaussian (EMG) distribution has the form

$$\begin{array}{l} \;\text{EMG}\;\left(\tau_{k};\lambda ,\sigma^{2}\right)=\frac{1}{2}\lambda_{j}e^{-\lambda_{j}\left(\tau_{k}-\frac{1}{2}\sigma^{2}\lambda_{j}\right)}\\ \;\times \left(1+erf\left(\frac{\tau_{k}-\lambda_{j}\sigma^{2}}{\sqrt{2}\sigma }\right)\right). \end{array}$$

**Fig. 1.**
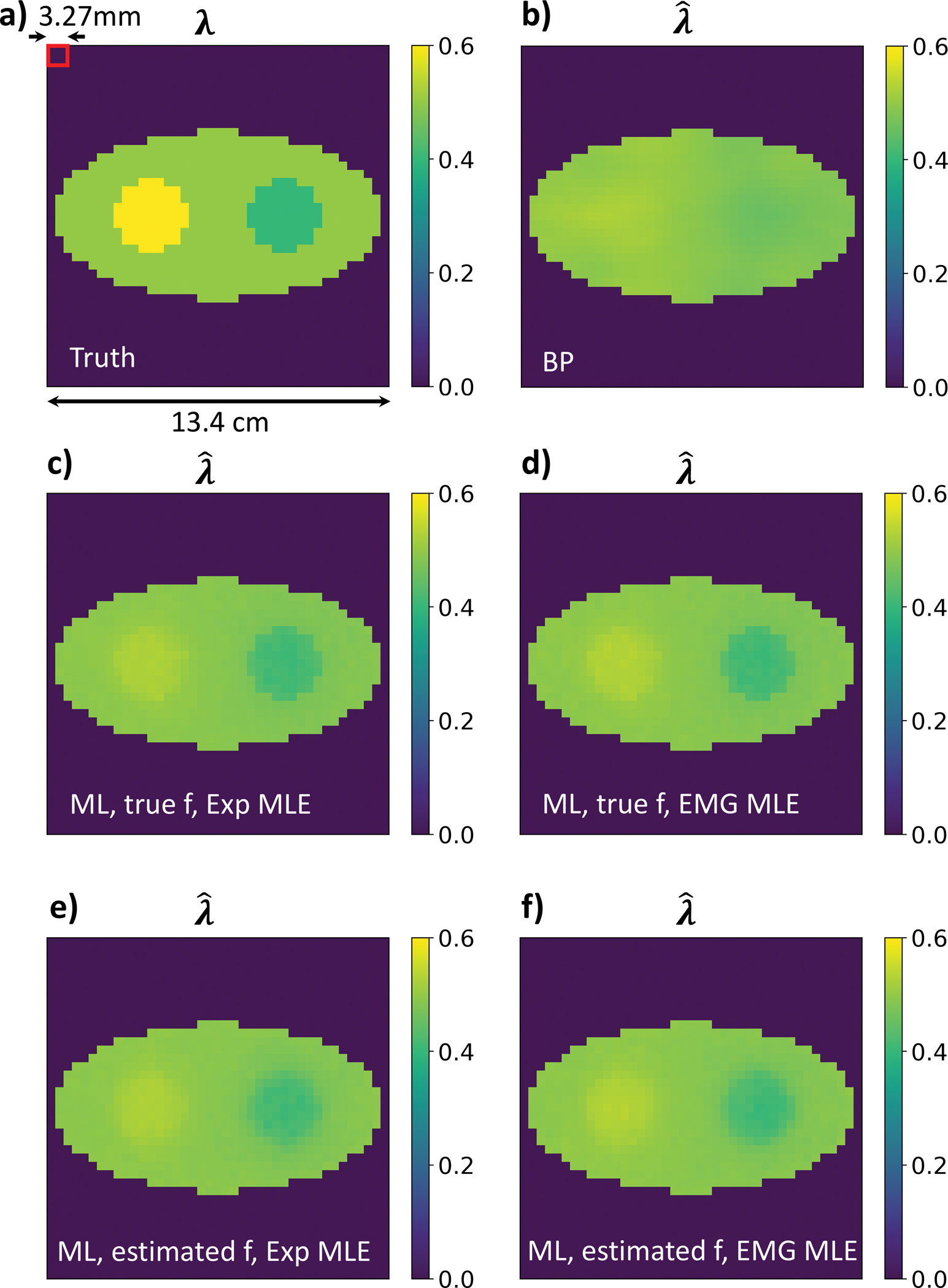
T(A) True λ image; (B) the BP estimate; (C) the Exp-MLE using true f and Exponentially distributed lifetime decays; (D) the EMG-MLE using true f; (E) the ExpMLE using estimated f; (F) the EMG-MLE using the estimated f. (Unit for λ and $\overset{ˆ}{\lambda }$: ns^−1^.).

The ML estimates of f were calculated using the maximum likelihood expectation maximization method (MLEM) [16]. The ML estimates of λ were obtained using an iterative gradient-based method with the step size equal to 2·10^−6^. We experimented with various step size values and observed that the step size was negatively correlated with the number of iterations required to achieve convergence but it did not affect the reconstructed results. The PLI data simulation procedure follows [12]. We conducted computer simulation studies for a 2-d TOF-PET system and simulated PLI events using Monte Carlo methods for a scanner with 288 detectors on a 57 cm diameter ring and a 570 ps coincidence resolving time (CRT), whose configuration parameters are close to existing clinical TOF-PET systems. Phantom 1 in [12] was used, which has a uniform elliptical activity image measuring 13.0 cm and 6.5 cm along the major and minor axes, respectively. Its rate constant image (Fig. 1A.) contains two discs that have different λ values (0.4 and 0.6 ns^−1^) from the background ellipse (0.5 ns^−1^), and the remaining pixels have λ = 0 ns^−1^. We call the elliptical region where λ > 0 the region of interest (ROI). The images are discretized into square pixels of 3.27 × 3.27 mm^2^, with 41 × 41 pixels.

To evaluate the quality of the reconstructed lifetime images, we adapted the contrast recovery coefficient (CRC) and background variability (BV) as outlined in [17] from count-data-based metrics to their counterparts in lifetime data.

The CRC is defined as

(8) $${CRC}_{p}=\frac{{\overset{ˆ}{\lambda }}_{p}/{\overset{ˆ}{\lambda }}_{B}-1}{\lambda_{p}/\lambda_{B}-1},p=1,2$$

where $\lambda_{p}$ is the average rate constant across the pixels in disc p in the reconstructed image, ${\overset{ˆ}{\lambda }}_{B}$, is the average rate constant across the pixels in the background in the reconstructed image, ${\overset{ˆ}{\lambda }}_{p}$ is the true rate constant for disc $p\left(\lambda_{1}=0.6{\;ns}^{-1},\lambda_{2}=0.4{\;ns}^{-1}\right)$, and $\lambda_{B}=0.5\;{ns}^{-1}$ is the true rate constant for the background. The final CRC is calculated as the average of CRC_1_ and CRC_2_. The BV is defined as

(9) $$BV={\hat{SD}}_{B}/{\overset{ˆ}{\lambda }}_{B},$$

where ${\hat{SD}}_{B}$ is the standard deviation of the rate constant across the pixels in the background of the estimated image.

In addition, we quantify the reconstruction accuracy with the normalized mean square error (NMSE), calculated as

(10) $$\text{NMSE}=\frac{\|\overset{ˆ}{\lambda }-\lambda {\|}^{2}}{\|\lambda {\|}^{2}},$$

where $\overset{ˆ}{\lambda }$ and λ are the reconstructed and ground-truth images, respectively, and II· II is the L-2 norm.

To ensure the convergence of the iterative algorithm for λ reconstruction, we introduced a termination criterion to determine the number of iterations, defined by

(11) $$\text{rdiff}=\frac{\left|{NMSE}_{i}-{NMSE}_{i-1}\right|}{{NMSE}_{i-1}},$$

which is the relative difference in NMSE between iteration i and iteration i-1. When the relative difference is smaller than a pre-specified tolerance value, the iteration will be terminated. With the implementation of this termination criterion, the number of iterations is not fixed. In our simulations, it ranges from 500 to 1500 and is negatively correlated with the event size used.

We implemented the proposed method using combinations of the following simulation settings:

1. Distribution assumption: i) Exponential distribution ii) EMG distribution.
2. f image used for λ reconstruction: true f ii) estimated f.
3. Timing uncertainty ($\sigma_{t}$) 0.085 ns ii) 0.16 ns iii) 0.242 ns iv) 0.5 ns.
4. Event size, calculated as the product of the number of pixels in the ROI (627 in the phantom used) and a multiplier of choice which represents the number of PLI events per pixel. i) 627 × 100 ii) 627 × 200 iii) 627 × 500 iv) 627 × 1000.

For each simulation setting, the proposed algorithm was repeated 20 times, yielding a reconstructed λ image in each replication. We calculate the mean of the 20 reconstructed λ images to obtain a final estimation and use the standard deviation to evaluate the variability of the estimation.

As in [13], ${\lambda_{j}}^{-1}$ was also estimated by back-projecting (BP) the events into pixels according to the system matrix H and then taking the average of $\tau \wedge_{k}$. For each pixel j,

(12) $${\left({\overset{ˆ}{\lambda }}_{BP}^{-1}\right)}_{j}=\frac{\sum_{k}\;H_{c_{k},j}\tau_{k}^{\prime }}{\sum_{k}\;H_{c_{k},j}}.$$

## RESULTS

### Exp-MLE *versus* EMG-MLE

Fig. 1. compares images obtained by BP, the MLE using an Exponential distribution for the lifetime decays (denoted as ‘Exp-MLE’), and the proposed MLE using an Exponential modified Gaussian distribution for the lifetime decays (‘EMG-MLE’). For each method, 627 × 100 events were generated, and both true f and estimated f were applied. In each setting, 20 independent Monte Carlo instances were generated to achieve an averaged reconstructed λ. Fig. 1B. shows that ${\overset{ˆ}{\lambda }}_{BP}$ is significantly blurry and can barely reconstruct the two discs. Fig. 1C.–F. show that, regardless of the choice between Exp-MLE or EMG-MLE, and whether using the true f or the estimated f, the proposed algorithm can well identify the edges of the region of interest (ROI) and the two discs. In the following results, unless otherwise specified, we used EMG-MLE and the estimated f image.

**Fig. 2.**
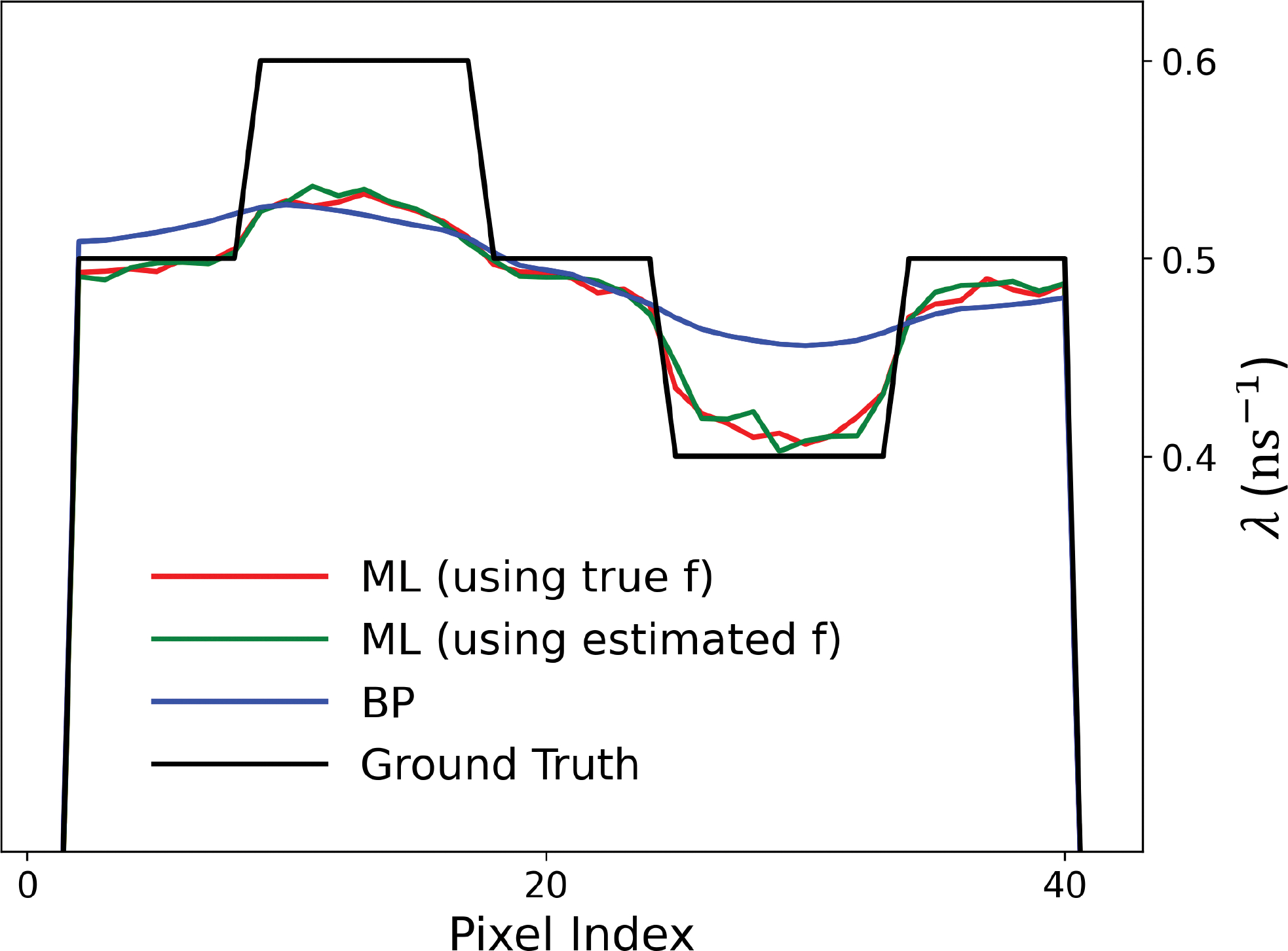
The cross-section through the horizontal centre of the reconstructed $\overset{ˆ}{\lambda }$ (red: the proposed EMG-MLE using the true f, green: the proposed EMG-MLE using the estimated f and blue: BP) and the true λ (black curve).

**Fig. 3.**
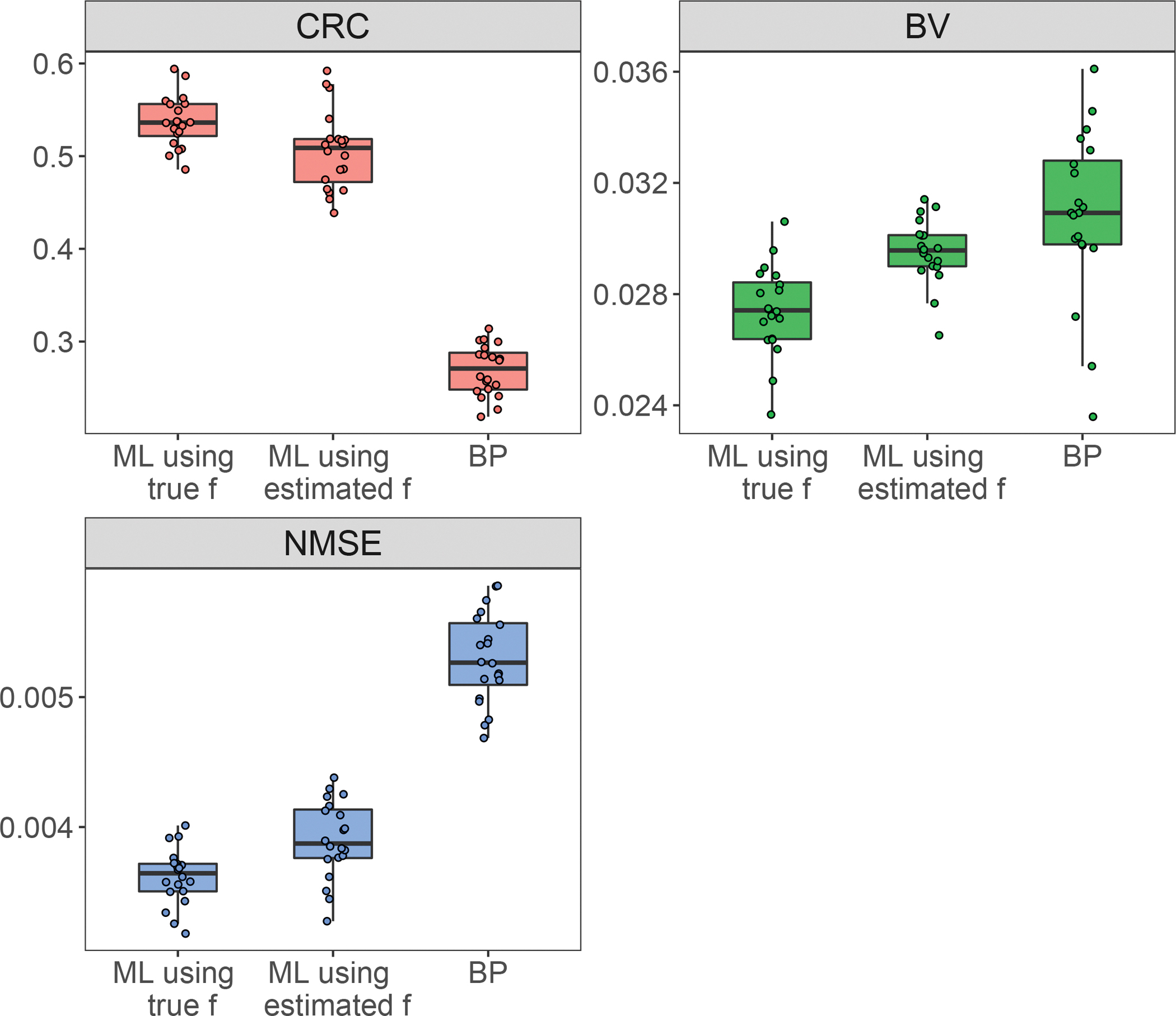
CRC, BV and NMSE for the 20 simulations of ML using the true and estimated f and BP.

Fig. 2. presents a comparison of the horizontal profiles across the centre of the ground-truth and reconstructed λ images. It highlights the agreement between the profile of ${\overset{ˆ}{\lambda }}_{ML}$ when using both the true f and the estimated f with the ground truth. Additionally, it demonstrates that the profile of ${\overset{ˆ}{\lambda }}_{BP}$ is nearly flat. Fig. 3. confirms that the ML-reconstructed lifetime images display superior contrast and higher sharpness compared to the BP method. This is evident in the higher CRC, lower BV and NMSE values achieved by the ML method, regardless of whether the ground truth or estimated activity image was used for lifetime reconstruction.

**Fig. 4.**
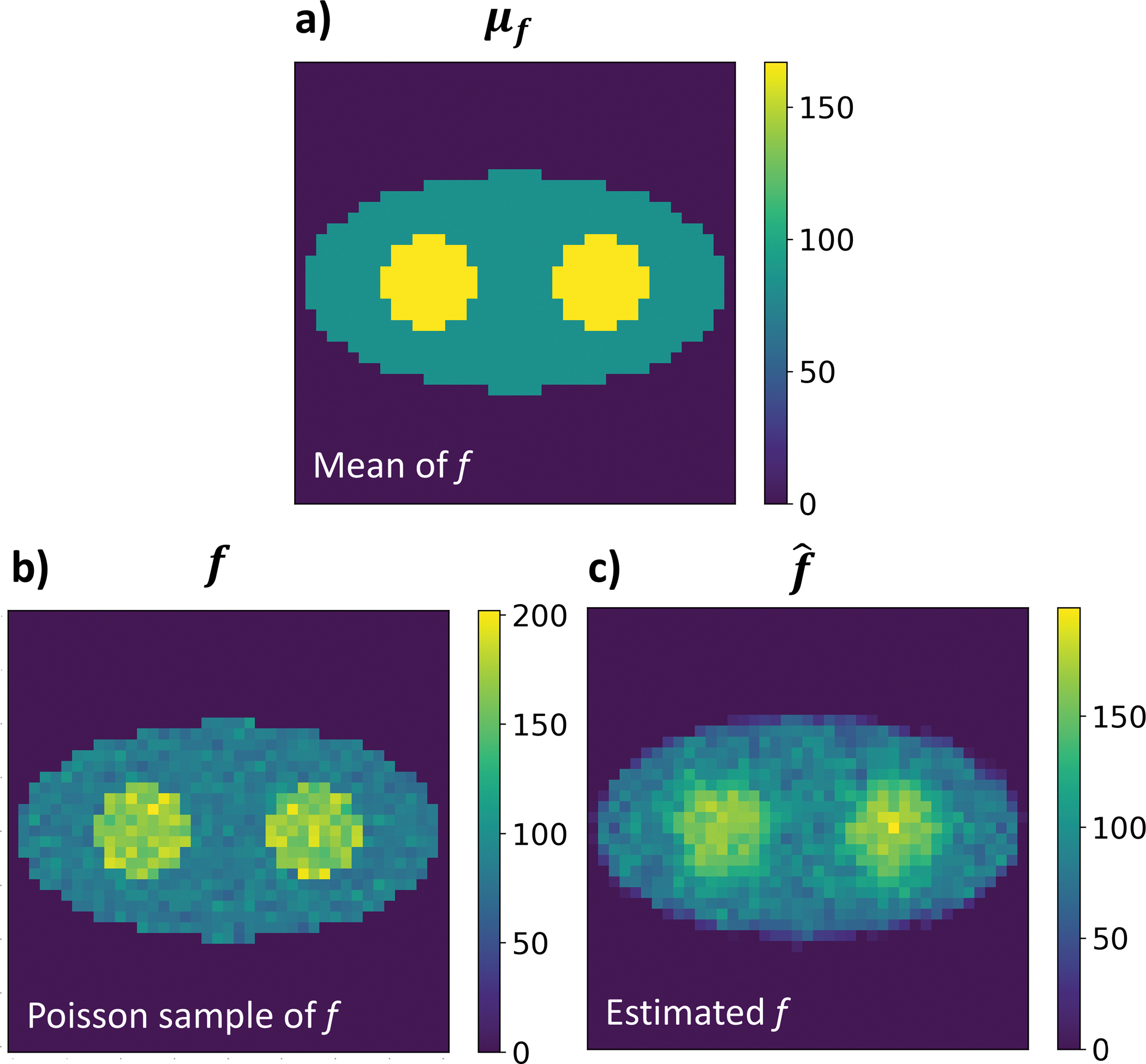
(A) The Poisson mean of the f image; (B) A Poisson sample of the f image; (C) The estimated f image. The event size is 100 per pixel. f=168 in the two discs and f=84 in the background (Unit: counts.).

Fig. 4. exhibits the ground truth (the Poisson mean) for the f map, a random Poisson sample based on the mean, and the estimated f map. It shows that the two discs can be clearly identified.

**Fig. 5.**
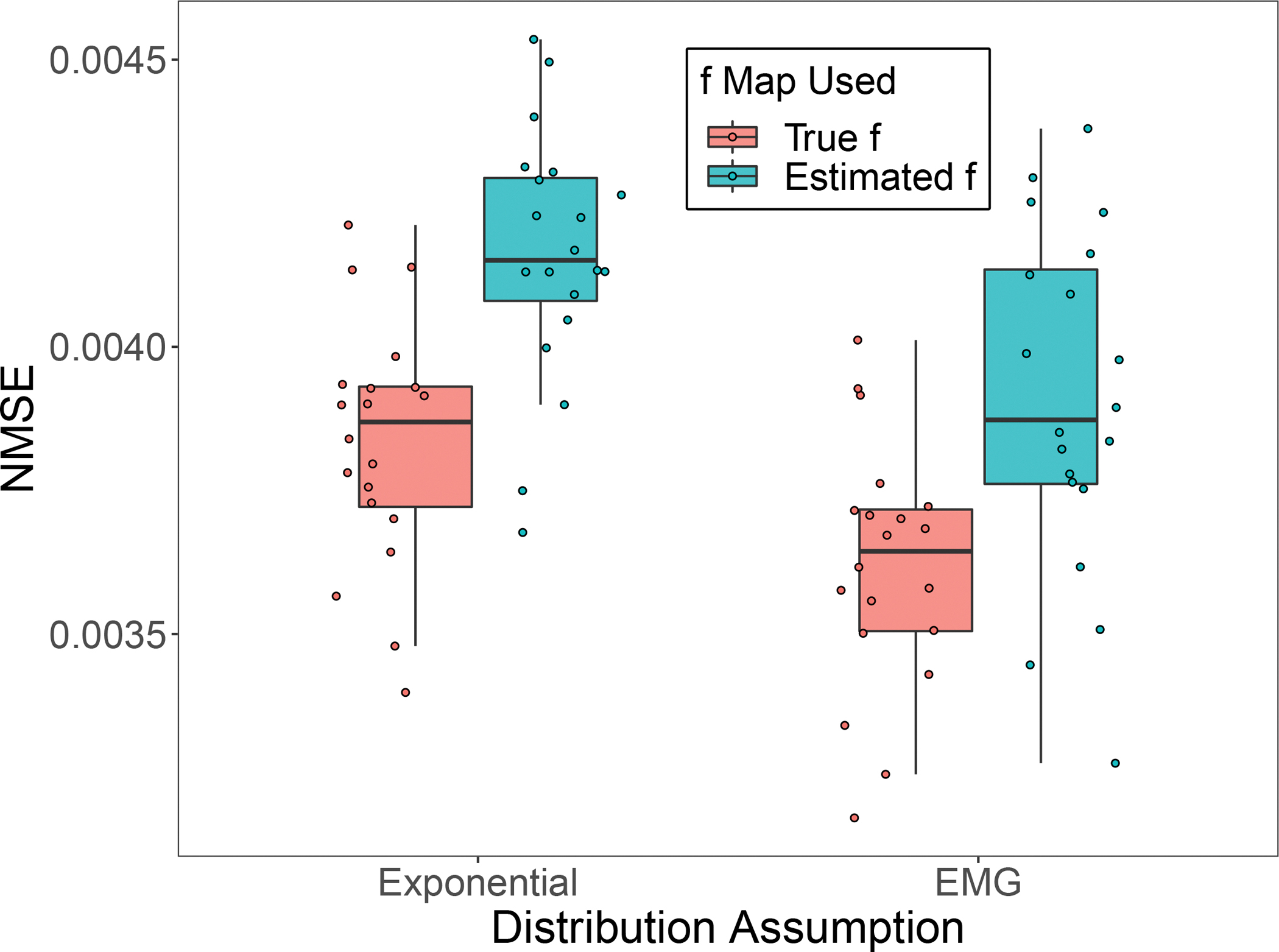
NMSE of the 20 replications of λ reconstruction using Exp-MLE and EMGMLE, and the true and estimated f image.

Fig. 5. indicates that the NMSE of Exp-MLE is slightly higher than that of EMG-MLE, implying that the EMG model better captures the estimated lifetime blurred by a Gaussian error.

**Fig. 6.**
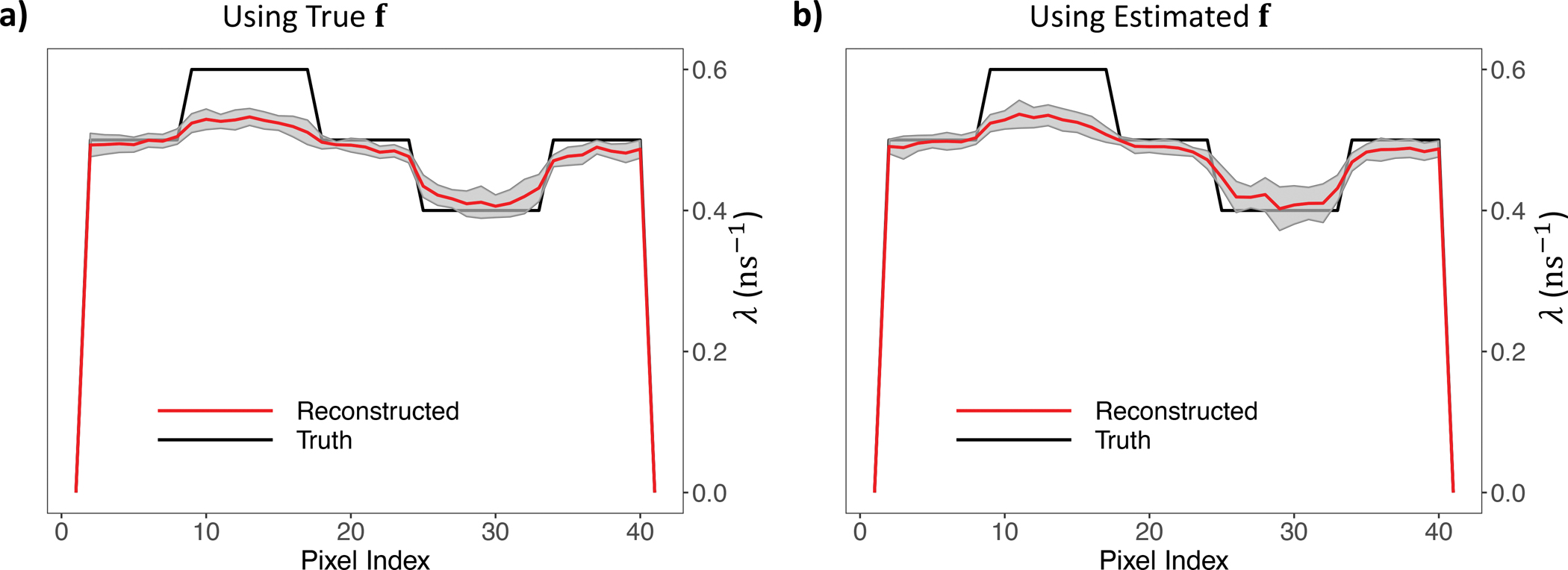
Horizontal profile across the centre of ${\overset{ˆ}{\lambda }}_{ML}$ and the true λ. The grey shaded region represents the ±1 SD range of the estimation for the 20 replications. (A) True f was used for λ reconstruction; (B) Estimated f was used for λ reconstruction.

Fig. 6. displays the horizontal profile across the centre of the reconstructed and the ground-truth lifetime image. The shaded region represents the ± one standard deviation range of the reconstructed lifetime at the corresponding image pixel across 20 simulations. It illustrates that employing the estimated activity image for lifetime image reconstruction can attain nearly identical accuracy and stability as using the true activity image. This underscores the high applicability of our method in real-life scenarios where the true activity image is unavailable.

### PLI Reconstruction Using Different Timing uncertainties

**Fig. 7.**
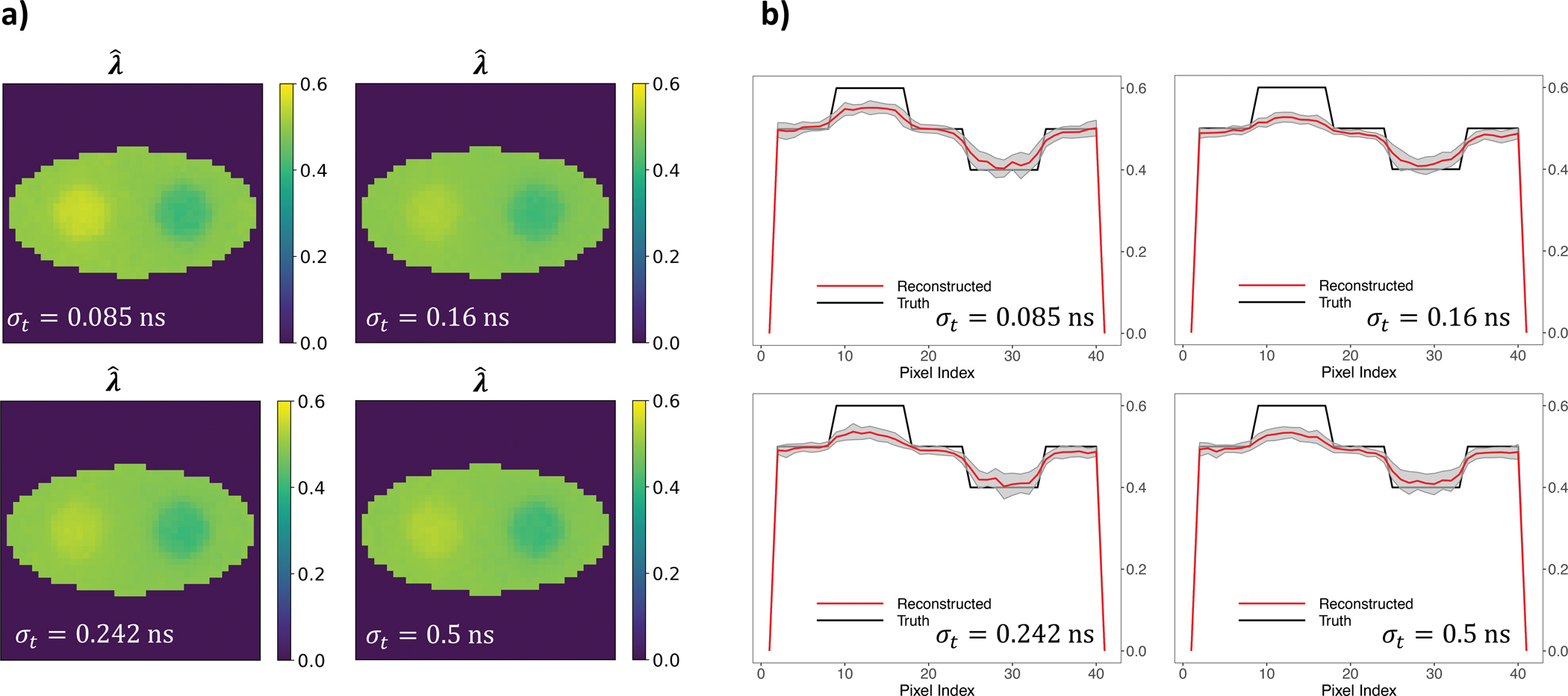
(A) ${\overset{ˆ}{\lambda }}_{ML}$ (unit: ns^−1^) using different $\sigma_{t}$ values; (B) Horizontal profiles across centre of the ${\overset{ˆ}{\lambda }}_{ML}$ (unit: ns^−1^) using different $\sigma_{t}$ values.

**Fig. 8.**
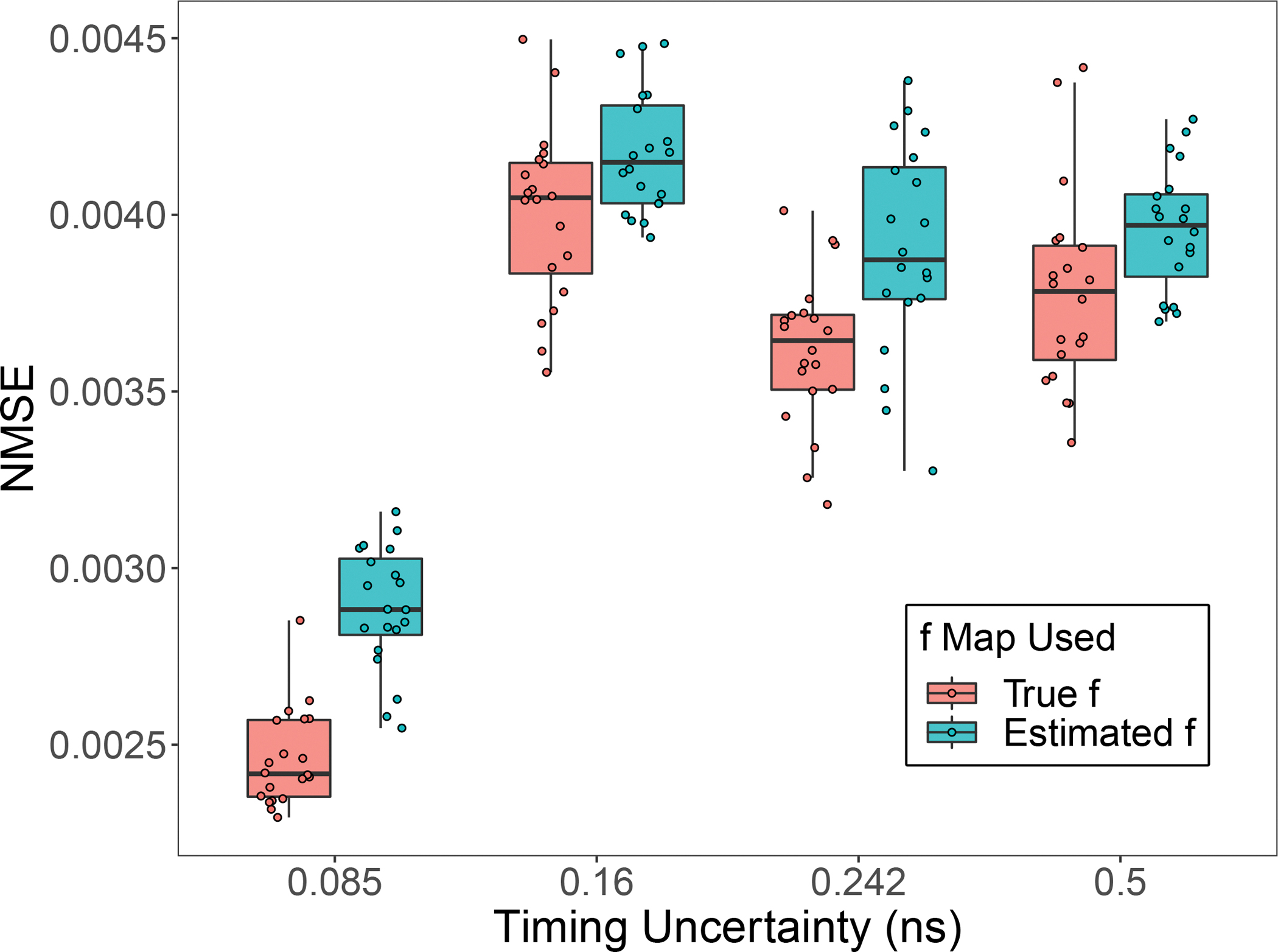
NMSE of the 20 replications λ reconstruction using different timing uncertainties.

The default value of

$$\sigma_{t}=\frac{0.57}{2\sqrt{2ln(2)}}=0.242\;ns$$

characterises the performance of the EXPLORER whole-body scanner. To assess the impact of various CRTs on our ML-based reconstruction method, we conducted reconstructions using simulated events with different $\sigma_{t}$ values: 0.16 ns, 0.242 ns, 0.5 ns, 1 ns, and 2 ns. The same $\sigma_{t}$ values were utilised in both the simulations and the EMG decay model. We kept the total number of events constant at 627 · 100 and maintained 20 replications for each simulation setting. Fig. 7. displays the average reconstructed decay rate constant images along with horizontal profiles, including the ±1 standard deviation range of the λ reconstruction. Meanwhile, Fig. 8. exhibits the NMSE across the 20 replications for varying timing uncertainties. The NMSE of timing uncertainty at 0.085 ns is significantly lower than the other three σt values, suggesting that lower time resolution does improve the reconstruction results. This highlights the importance of time resolution, indicating that a smaller σt can lead to improved reconstruction quality.

### Reconstruction Using Different Event Sizes

Both Qi and Huang [13] and our previous work [12] used over 1 million simulated PLI events, resulting in more than 2,000 PLI events per ROI pixel. In order to investigate the sufficient number of events per pixel needed to achieve accurate reconstruction of the decay rate constant, we substantially lowered the number of events pixels to 100 and subsequently experimented with a sequence of numbers of events per pixel, ranging from 200 to 1,000.

**Fig. 9.**
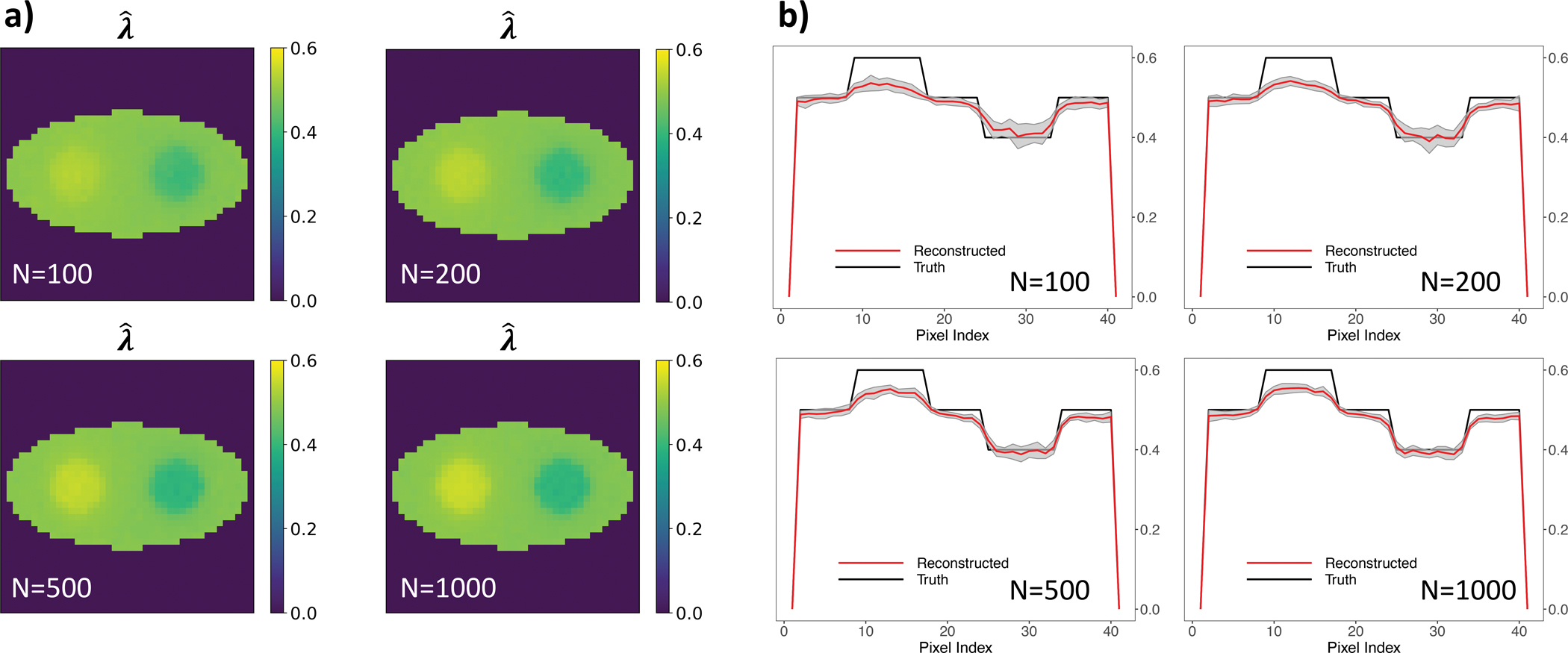
(A) ${\overset{ˆ}{\lambda }}_{ML}$ (unit: ns^−1^) using different numbers of events per pixel; (B) Horizontal profiles across center of the (A) ${\overset{ˆ}{\lambda }}_{ML}$ (unit: ns^−1^) using different numbers of events per pixel.

The averaged reconstructed λ images, along with their respective horizontal profiles displaying a ±1 standard deviation range, are depicted in Fig. 9. The results illustrate an enhancement in both accuracy and consistency of the λ image reconstructions with a larger number of events. In addition, even with as few as uncertainties. 100 events per pixel, we can still attain a highly precise reconstructed decay rate constant image with an exceptionally small standard deviation, demonstrating the robustness of the method.

**Fig. 10.**
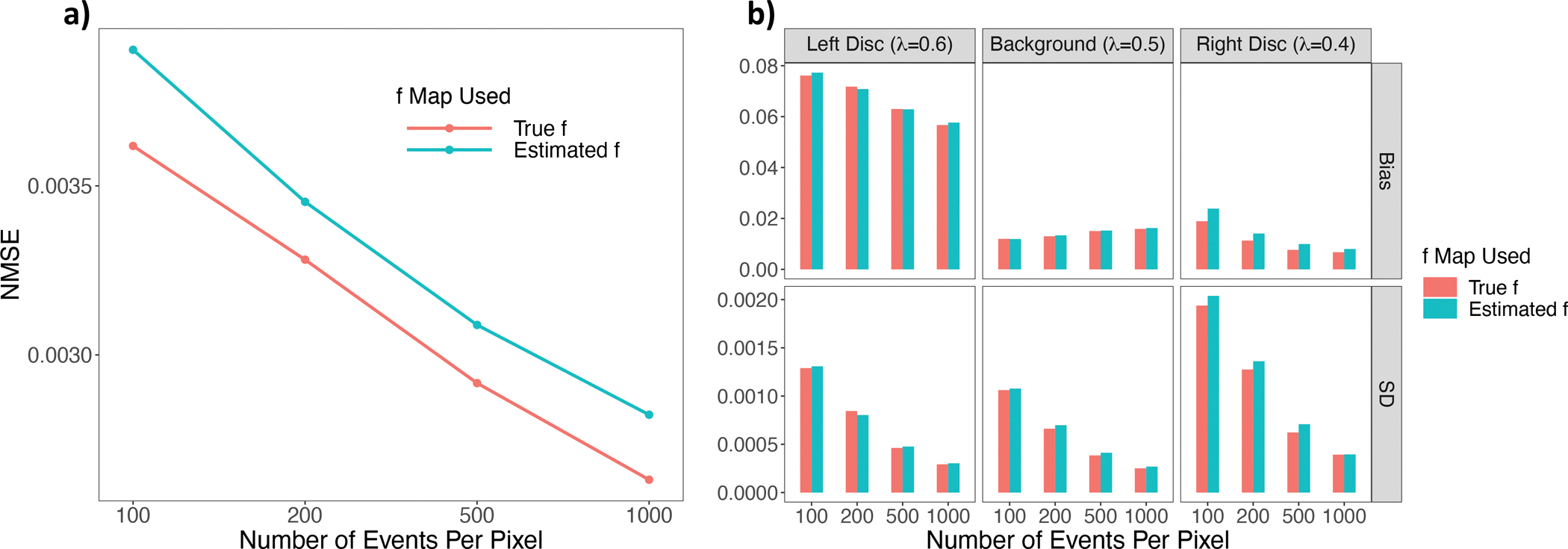
(A) NMSE for different number of events per pixel, averaged from 20 simulations; (B) Bias and standard deviations of the ML estimation for the three separate regions of interest, averaged from 20 simulation experiments.

The NMSEs across 20 simulations for varying event counts per pixel are presented in Fig. 10A. As the number of events per pixel decreases, the NMSE exhibits a consistent decrease, with notably higher NMSE values when using the estimated activity image. To further investigate the performance of the ML reconstruction, we separate the three regions of interest – the left disc with λ = 0.6 ns^−1^, the background with λ = 0.5 ns^−1^ and the right disc with λ = 0.4 ns^−1^, and compute the bias and standard deviation of the ML estimation of these regions.

Fig. 10B. shows that aside from the background, where the bias remains relatively stable across different event sizes, both bias and standard deviation exhibit a consistent reduction as the sample size increases. Additionally, employing the true activity image tends to yield lower bias and standard deviation values. Furthermore, we observe a trade-off between bias and standard deviation. For instance, the left disc demonstrates higher bias compared to the right disc, but it concurrently exhibits lower standard deviation. This underscores the nuanced relationship between these two evaluation metrics.

## CONCLUSION AND DISCUSSIONS

Based on the ML algorithm for reconstructing the positronium lifetime image developed in [12], in this paper we further validated the performance and the applicability of the method through simulation studies across a range of settings. The reconstructed lifetime images consistently exhibited good contrast and sharpness while maintaining quantitative accuracy despite the presence of uncertainty in time measurements. The proposed method proved robust, yielding satisfactory results under varying scenarios.

The estimation stability of the proposed method was evaluated by computing the standard deviation of estimates. The results indicated stability in the ML reconstruction algorithm. Furthermore, our approach was able to produce accurate lifetime reconstructions when the event size was reduced to 100 events per pixel. This finding highlights the practicality and efficiency of our proposed method.

In this article our simulated data only included decay events for ortho-positronium. For future research endeavours, we plan to extend the proposed method to a two-component decay model, incorporating the annihilation of both long-lived ortho-positronium and short-lived para-positronium. Additionally, our simulations have been limited to 2D TOF PET data. We will explore the development of algorithms to enable the reconstruction of 3D images. Furthermore, we aim to apply the proposed method to real data, subjecting it to practical scenarios to investigate its applicability and validate its performance in real-world contexts.
